# Antiretroviral Therapy Normalizes Autoantibody Profile of HIV Patients by Decreasing CD33^+^CD11b^+^HLA-DR^+^ Cells: A Cross-Sectional Study

**DOI:** 10.1097/MD.0000000000003285

**Published:** 2016-04-18

**Authors:** Zhefeng Meng, Ling Du, Ningjie Hu, Daniel Byrd, Tohti Amet, Mona Desai, Nicole Shepherd, Jie Lan, Renzhi Han, Qigui Yu

**Affiliations:** From the Oncology Bioinformatics Center, Minhang Hospital, Fudan University (ZM, LD); Shanghai, China; Department of Microbiology and Immunology and Center for AIDS Research, Indiana University School of Medicine, Indianapolis, Indiana, USA (ZM, DB, TA, NS, JL, QY); Zhejiang Provincial Key Laboratory for Technology and Application of Model Organisms, Wenzhou Medical University, University Park, Wenzhou, China (NH); Division of Infectious Diseases, Department of Medicine, Indiana University School of Medicine, Indianapolis, IN (MD, SG, QY); and Department of Surgery, Davis Heart and Lung Research Institute, Biomedical Sciences Graduate Program, Biophysics Graduate Program, The Ohio State University Wexner Medical Center, Columbus, OH, USA (RH).

## Abstract

Autoimmune manifestations are common in human immunodeficiency virus (HIV) patients. However, the autoantibody spectrum associated with HIV infection and the impact of antiretroviral therapy (ART) remains to be determined.

The plasma autoantibody spectrum for HIV patients was characterized by protein microarrays containing 83 autoantigens and confirmed by enzyme-linked immunosorbent assay (ELISA). Regulatory T cells (Tregs) and myeloid-derived suppressor cells (MDSCs) were analyzed by flow cytometry and their effects on autoantibodies production were determined by B cell ELISpot.

Higher levels of autoantibody and higher prevalence of elevated autoantibodies were observed in ART-naive HIV patients compared to healthy subjects and HIV patients on ART. The highest frequency of CD33^+^CD11b^+^HLA-DR^+^ cells was observed in ART-naive HIV patients and was associated with the quantity of elevated autoantibodies. In addition, CD33^+^CD11b^+^HLA-DR^+^ cells other than Tregs or MDSCs boost the B cell response in a dose-dependent manner by *in vitro* assay.

In summary, HIV infection leads to elevation of autoantibodies while ART suppresses the autoimmune manifestation by decreasing CD33^+^CD11b^+^HLA-DR^+^ cells *in vivo*.

The roles of CD33^+^CD11b^+^HLA-DR^+^ cells on disease progression in HIV patients needs further assessment.

## INTRODUCTION

Increased prevalence and titer of circulating autoantibodies have been reported in patients infected with human immunodeficiency virus (HIV) type 1.^1^ Up to 68.1% of HIV patients have circulating antinuclear antibodies (ANAs),^1–4^ including autoantibodies against cellular DNA,^1,3^ small nuclear ribonucleoproteins (snRNPs),^1,5^ histone H2B,^6^ Smith (Sm),^7^ SSA (Ro),^8^ and SSB (La).^8^ In addition to ANAs, autoantibodies against peripheral blood cells,^7^ MHC II,^7^ and phospholipids^3,7,9^ have also been frequently observed in HIV patients. The increased incidence and titer of autoantibodies are associated with lower CD4^+^ T cell count and higher mortality in HIV patients who are naive to antiretroviral therapy (ART),^3^ indicating that autoantibodies may play a pathogenic role or can serve as a prognostic indicator in HIV infection. In fact, autoimmune diseases including systemic lupus erythematosus (SLE), rheumatological syndromes, antiphospholipid syndrome, vasculitis, primary biliary cirrhosis, polymyositis, Graves’ disease, and idiopathic thrombocytopenic purpura have been frequently reported in HIV/acquired immune deficiency syndrome (AIDS) patients.^1^ Therefore, it is important to characterize the spectrum of emerging autoantibodies in HIV patients as this may shed light on resolving HIV infection related autoimmune manifestation.

Moreover, in the current era of ART, a plethora of concerns about autoimmune manifestations in HIV patients have been raised, which could determine the therapeutic efficiency. Therefore, further elucidating the impact of ART on autoantibody spectrum in HIV patients is important and valuable for therapeutic evaluation.

Recently, immune regulatory cells, including regulatory T cells (Tregs, CD25^+^FoxP3^+^) and myeloid-derived suppressor cells (MDSCs, CD33^+^CD11b^+^HLA-DR^−^), are widely observed to increase in frequency in HIV patients, which is thought to be responsible for the severe impaired cytotoxic T cell (CTL) immune response *in vivo*.^10–16^ These immune regulatory cells play pivotal roles in the maintenance of self-tolerance as well as control of immune activation, particularly during chronic infections.^17–23^ In the setting of autoimmune manifestation caused by HIV infection, it also becomes crucial to know the roles of Tregs, MDSCs, and other immune cells in the development of an autoimmune response.

In the present study, we investigated autoantibody profiles in HIV patients who had maintained undetectable viral loads on long-term ART, HIV patients who were ART-naïve, and healthy subjects, and further explored the roles of immune regulatory cells on regulating the autoimmune response.

## METHODS

### Study Participants

The study was performed according to a protocol approved by the Institutional Review Boards for Human Research at the Indiana University School of Medicine (Indianapolis, IN). Written informed consent was obtained from each of all participants before blood collection. Three groups of individuals were recruited in this study: 20 healthy controls, 17 HIV-infected ART-naive individuals (CD4 cell counts: 422 cells/mm^3^, range 16–1237; viral load: 11,532 copies/mL, range 59–570,156 copies/mL), and 14 HIV-infected individuals on ART with undetectable viral load (<50 copies/mL) for at least 2 years (CD4 cell counts: 556 cells/mm^3^, range 231–1310). Peripheral blood from these participants was collected in BD Vacutainer tubes (containing 143 USP units of sodium heparin per 10-mL tube, BD, Franklin Lakes, NJ). These blood samples were separated into peripheral blood mononuclear cells (PBMCs) and plasma and stored at **−**80 °C or liquid nitrogen until use.

### Autoantigen Protein Microarray

Circulating autoantibody profiles in plasma samples from uninfected and HIV-infected individuals were determined using an autoantigen protein microarray as previously described.^24,25^ The autoantigen protein microarray included 83 autoantigens and 4 control proteins and was performed in the Microarray Core Facility of the Texas University (Dallas, TX). Briefly, the plasma sample diluted at 1:100 was added to the arrays in duplicate. Net fluorescence intensities (NFIs) were generated by A Genepix 4000B scanner and Genepix Pro 6.0 software and normalized using anti-human IgG spotted onto each array. Values obtained from duplicate spots were averaged. Tests of significance between groups were carried out using a Student's *t*-test or one-way ANOVA for multiple groups (GraphPad Prism 5.02). Correlations between continuous variables were determined using Pearson *r*, and dichotomized variables were compared using Fisher exact test. A *P* value < 0.05 was considered significant. Diagrams with row-wise and column-wise clustering were generated using Cluster and Treeview software (http://rana.lbl.gov/EisenSoftware.htm).

### ELISA

An enzyme-linked immunosorbent assay (ELISA) was developed to confirm the microarray data and to titrate autoantibodies in plasma samples. Briefly, 96-well plates were coated with individual recombinant human autoantigens including centromere protein B (CENP-B), Intrinsic Factor, nuclear pore glycoprotein-210 (gp210), mitochondrial antibody subtype M2 (MA-M2), synthetase (PL7), proteins of the nucleolar PM/Scl macromolecular complex (PM/Scl-75), SP100, signal recognition particle 54 kDa protein (SRP54), Lupus La protein or Sjögren syndrome type B antigen (La/SS-B), and small nucleoprotein particles (snRNPs) such as U1-snRNP-68, U1-snRNP-A, U1-snRNP-BB′, and U1-snRNP-C (SurModics, Eden Prairie, MN). After washing and blocking with 5% FBS/PBS, plates were incubated with serially diluted plasma, followed by the addition of goat anti-human IgG monoclonal antibody conjugated with horseradish peroxidase (HRP). Optical densities (OD) at 450 nm were determined using an ELISA plate reader (ELX 808 microplate reader, Winooski, VT). Results were expressed as titers. All ELISA reagents were purchased from SurModics (Eden Prairie, MN). The observed endpoint titer for the autoantibody assay was the highest plasma dilution that yielded an OD greater than the value that defined the cutoff between positive and negative results.

### PBMC Preparation, Cell Depletion, and Sorting

PBMCs were isolated from whole blood by Ficoll centrifugation and analyzed immediately or cryopreserved at −80 °C. PBMCs were subjected to a positive selection of CD33^+^CD11b^+^ or CD3^+^CD4^+^CD25^+^cells by Fluorescence Activated Cell Sorting (FACS) using a BD FACS Aria (BD Biosciences, San Jose, CA). After sorting CD33^+^CD11b^+^ or CD3^+^CD4^+^CD25^+^ cells, the remaining depleted PBMCs were also harvested for further usage. The isolated CD33^+^CD11b^+^ cells were further sorted into CD11b^+^HLA-DR^+^ or CD11b^+^HLA-DR^−^ by FACS using a BD FACSAria (BD Biosciences, San Jose, CA).

### Flow Cytometric Analysis

Cell surface staining with antibodies conjugated with fluorochromes was performed as previously described.^26^ The following anti-human antibodies conjugated with fluorochromes were purchased from eBiosciences (San Diego, CA): CD14-FITC, CD4-FITC, CD11b-PE, CD25-PE, CD3-PerCP, CD33-PercpCY5.5, HLA-DR-APC, FoxP3-APC, and isotype-matched control antibodies conjugated with fluorochrome. Intracellular staining (ICS) with anti-human FoxP3-PE was performed using the FoxP3 staining buffer set (eBiosciences, San Diego, CA) according to the manufacturer's instructions. As a heterogeneous cell population, human MDSCs could be further divided into 2 subsets, monocytic (M-MDSC, CD14^+^) and granulocytic (G-MDSC, CD14^−^/CD15^+^).^12,18,20–23^ Given that G-MDSCs are unavailable in Ficoll-prepared PBMCs, we set the gating strategy for M-MDSCs: CD33^+^CD11b^+^/CD14^+^HLA-DR^Low^. Meanwhile, the gating strategy for Tregs was CD3^+^CD4^+^CD25^+^FoxP3^+^. Cells were collected on a FACSCalibur (BD). The data were analyzed using FlowJo software (TreeStar, San Carlos, CA). Appropriate isotype controls were used at the same protein concentration as the test antibodies, and control staining was performed during every FACS.

### B Cell ELISpot Assay

B cell ELISpot kit from MABTECH (Cincinnati, OH) was used to enumerate the number of autoantibody-secreting B cells based on the manufacturer's instructions. Briefly, 96-well plates with PVDF membrane were coated with 13 mixed autoantigens (4 μg/mL of each autoantigen) or anti-human IgG (15 μg/mL, MABTECH) after membrane activation with 70% ethanol. PBMCs or CD33^+^ cell-depleted PBMCs from HIV patients or healthy donors were stimulated with R848 (1 ng/mL)/IL-2 (10 ng/mL), the CD33^+^ cell-depleted PBMCs were cocultured with autologous sorted CD33^+^ cells at various ratios (1:1, 1:5, 1:10) in 24-well plates for 3 days. After harvesting the supernatant from cultured cells, these cells were washed 3 times and transferred into 96-well PVDF plates at 2.5 × 10^5^ cells/well for overnight culturing. Reactions were visualized using alkaline phosphatase (AP)-conjugated Streptavidin (MABTECH) and BCIP/NBT substrate (MABTECH). The number of spots per 10^6^ PBMCs, which represented the number of autoantibody-producing B cells, was calculated by an ELISpot plate reader (Bio-Sys GmbH, Karben, Germany).

### Statistical Analysis

Data were expressed as mean ± standard deviation (SD). Statistical differences were determined by paired Student's *t*-test for paired comparisons, one-way ANOVA with post-tests for multiple group comparisons and linear regression analysis for correlations using Prism 5.02 (Graphpad Software, La Jolla, CA). A *P* value less than 0.05 was considered to be significant. The data produced by the current ProtoArray platform were evaluated for the presence or absence of a significant signal, which is a commonly used approach for data analysis when using ELISA kits for autoantibody measurement. The significantly elevated antibodies were determined by *P* < 0.05 based on one-way ANOVA test among multiple groups.

## RESULTS

### Autoantibody Profiles Altered in HIV Patients

**FIGURE 1:**
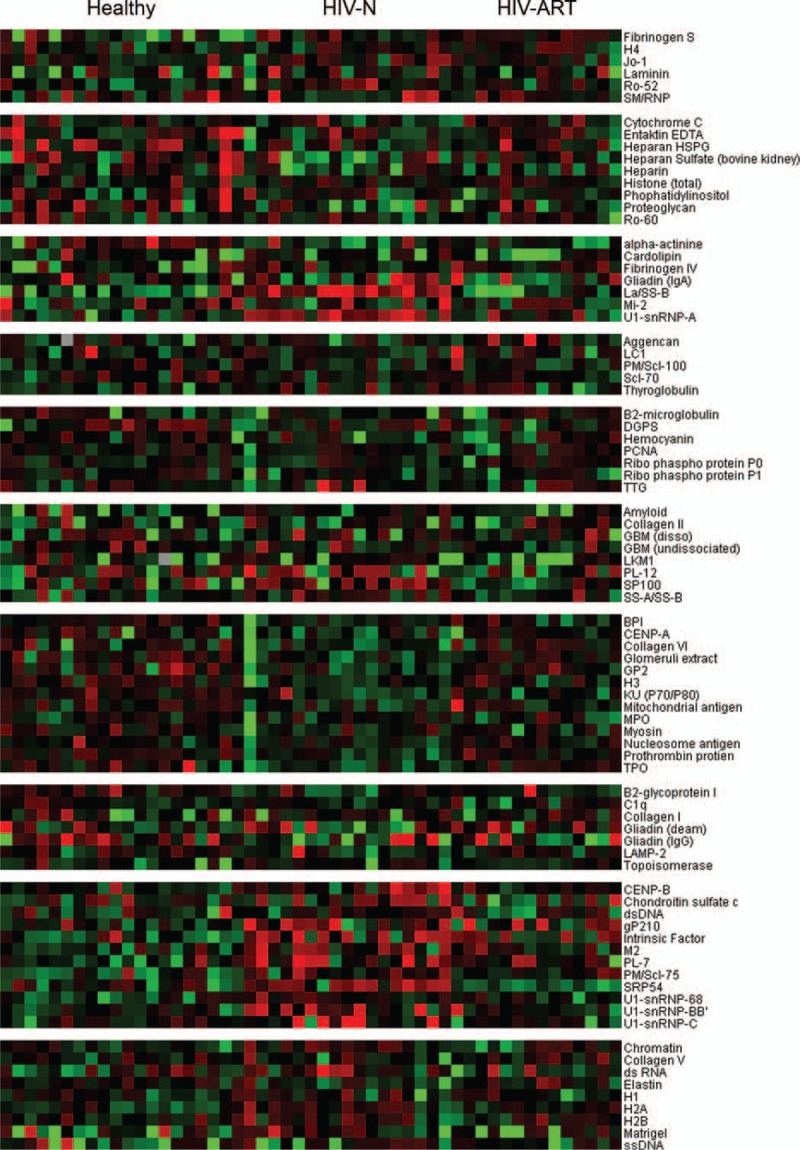
Heat map of the 83 IgG autoantibody reactivities in plasma samples. The average signal intensity of each antigen in each sample was normalized to the average intensity of total IgG which was printed in 6 replicates on the arrays as an internal control. The NFI data were used to generate the heat map. For each Ag, the reactivity intensities are depicted on a relative scale, where reactivities above the mean of all samples are colored red, reactivities below are colored green and reactivities close to the mean are black. Missing data were denoted in gray. The left margin indicates 10 distinct clusters of Ags with reactivities that clustered together in the tested samples. Healthy, healthy controls; HIV-N, HIV patients who were ART-naïve; HIV-ART, HIV patients on ART. Ag = antigen, ART = antiretroviral therapy, HIV = human immunodeficiency virus, NFI = net fluorescent intensity.

Eighty-three IgG autoantibody specificities across all plasma samples were measured via protein microarrays and analyzed using hierarchical clustering. Most clusters included antibodies which showed correlation to each other; 10 clusters could be distinguished from the IgG autoantibody heat map (Figure 1). Based on the microarray profiling, 15 antibodies (including CENPB, chromatin, collagen I, H2A, Intrinsic Factor, gp210, M2, PL7, PM/Scl-75, SP100, SRP54, La/SS-B, SS-A/SS-B, U1-snRNP-68, and U1-snRNP-A) were significantly elevated (*P* < 0.05) in ART-naive HIV patients compared to healthy controls (Table 1), which cluster together in clusters 2 and 7 (Figure 1). Eight of the 15 autoantibodies (including collagen I, gp210, H2A, Intrinsic Factor, M2, PL7, PM/Scl-75, and U1-snRNP-68) were also observed to be significantly elevated in the HIV patients on ART compared to those from healthy control subjects (cluster 7, Figure 1 and Table 1). When compared to HIV patients on ART, ART-naive HIV patients had 5 significantly elevated autoantibodies (gp210, H2A, SP100, PM/Scl75, and SRP54) (Table 1).

**TABLE 1:**
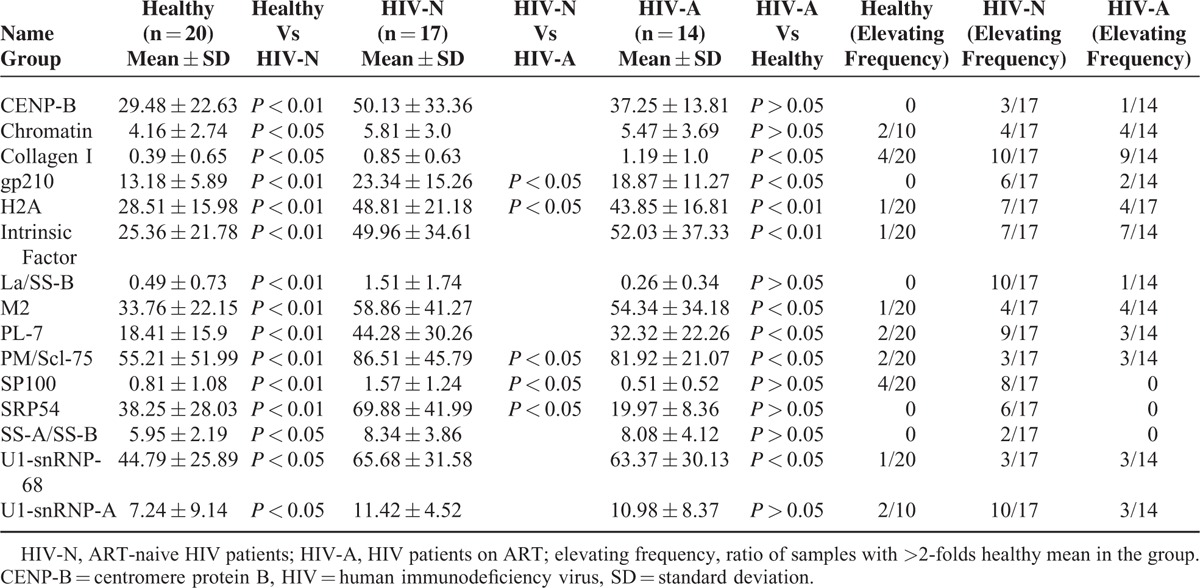
Elevated Autoantibodies in HIV Patients in Contrast to Healthy People

### HIV Patients Had Higher Prevalence of Elevated Autoantibodies Than Healthy Controls

**FIGURE 2:**
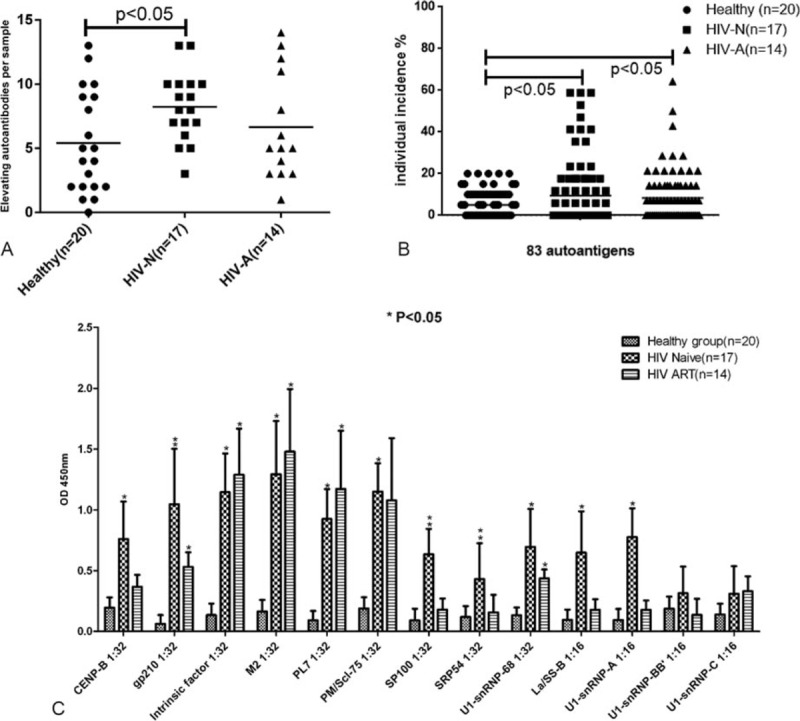
Comparative analysis of quantity and individual incidence of elevated autoantibodies in 3 groups and confirmation of varied autoantibodies levels detected by Microarray and by ELISA. (A) The quantity of elevated autoantibodies per sample in healthy groups, HIV patients without treatment, and HIV patients on ART. (B) Incidence of autoantibodies in healthy group, HIV patients without treatment and HIV patients with ART, respectively. (C) ELISA confirmation of plasma autoantibody against 13 autoantigens. Serial dilution was used to determine the final titer of autoantibodies in plasma, and 2-folds of the averaged negatives were considered as the cutoff. Data are presented as mean average ± SD for each of the antigens analyzed. A *P*-value less than 0.05 was considered statistically significant. ^∗^Significant difference between HIV patients and healthy control, ^∗∗^significant difference between HIV patients on ART groups and HIV patients without treatment as well as healthy control. ART = antiretroviral therapy, ELISA = enzyme-linked immunosorbent assay, HIV = human immunodeficiency virus, SD = standard deviation.

Mean NFIs of detecting autoantibody in healthy controls were set as cutoff values for evaluating the changes of autoantibodies: >2-fold cutoff as elevated autoantibody. Individually, HIV patients had a higher prevalence of elevated autoantibodies than healthy controls as indicated by the Heatmap (Figure 1). The number of elevated autoantibodies per sample ranged from 0 to 12 (mean = 5.4) in the healthy group, 3 to 13 (mean = 8.23) in ART-naive HIV patients, and 1 to 14 (mean = 6.64) in HIV patients on ART. Statistical analysis not only demonstrated that both ART-naive HIV patients and HIV patients on ART had a higher prevalence of elevated autoantibodies than healthy controls (*P* < 0.05, Figure 2A), but also revealed that HIV patients with ART-naive had a higher prevalence of elevated autoantibodies than those with ART (*P* < 0.05, Figure 2A).

### Higher Incidence of Individual Autoantibodies in HIV Patients

In order to understand the prevalence of a specific autoantibody in this population, the individual incidence of each autoantibody was calculated by the ratio of sample numbers with elevated autoantibody versus the total group size (Figure 2B). The common 32 autoantibodies did not show any elevation among all 3 groups. For the remaining 51 autoantibodies, the prevalence ranged from 0% to 20% (mean = 8.24%) in healthy controls, 0% to 58.82% (mean = 15.42%) in ART-naive HIV patients, and 0% to 50% (mean = 13.58%) in HIV patients on ART. In summary, both ART-naive HIV patients on ART showed significantly higher incidence of specific elevated autoantibody than healthy controls (*P* < 0.05, Figure 2B); ART-naive HIV patients presented with the highest prevalence of specific elevated autoantibody and was followed by HIV patients on ART. Furthermore, for the 15 specifically elevated autoantibodies, significantly higher titers and elevated frequency was observed in ART-naive HIV patients when compared to both healthy controls and HIV patients on ART (*P* < 0.05, Table 1). The microarray results established that ART-naive HIV patients had a higher incidence and higher titers of autoantibodies, and ART decreased the prevalence and titer of elevated autoantibodies per sample.

### Verification of Plasma Autoantibodies Against 13 Autoantigens Using ELISA

To verify the results obtained by the microarrays, we selected 13 antigens (CENPB, Intrinsic Factor, gp210, M2, PL7, PM/Scl-75, SP100, SRP54, La/SS-B, U1-snRNP-68, U1-snRNP-A, U1-snRNP-BB′, and U1-snRNP-C) and performed ELISA assays. In agreement with the microarray data, the ELISA data demonstrated that 11 out of the 13 autoantibodies in ART-naive HIV patients and 5 out of the 13 in HIV patients on ART were significantly higher than the healthy controls (Figure 2C). Additionally, 3 of 5 autoantibodies (gp210, H2A, SP100, PM/Scl75, and SRP54) in ART-naive HIV patients were identified to be higher than those of HIV patients on ART. Although no significant difference was observed between these two HIV patients groups, the PM/Scl-75 autoantibody in ART-naive HIV patients was significantly higher than the healthy control, while it was not increased in HIV patients on ART.

### Depletion of CD33^+^CD11b^+^ Cells Remarkably Impaired the Autoreactive B Cell Response *Ex Vivo*

**FIGURE 3:**
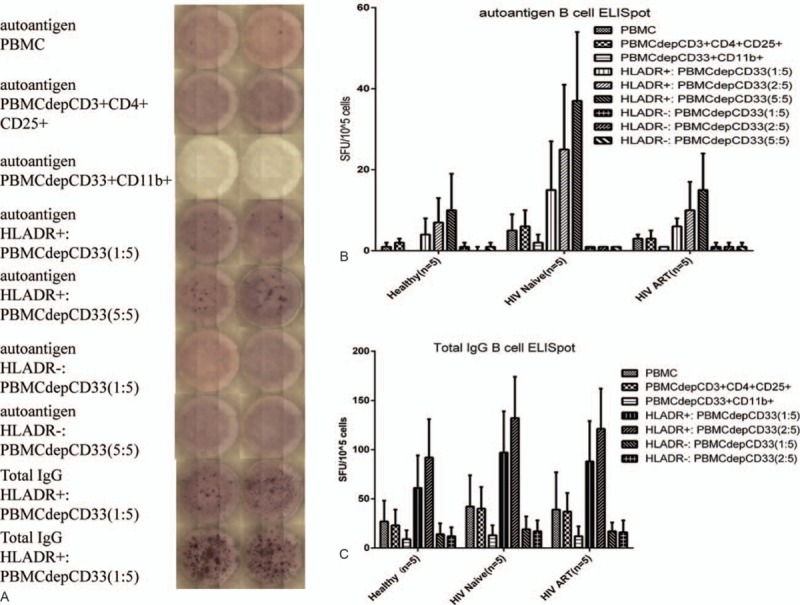
B cell ELISpot assay. PBMCs and CD33^+^ cell-depleted PBMCs were stimulated with R848 (1 ng/mL)/IL-2 (10 ng/mL) and cultured alone or cocultured with autologous isolated CD33^+^ cells at indicated ratios in 24-well plates for 3 days before performing ELISpot and cytokine detection. (A) Representative samples of ELISpot assay. Autoantigen specific assay was performed with 13 mixed autoantigens, while total IgG assays were performed with anti-human IgG. (B) Statistical analysis of autoantigen-specific circulating B cells in direct and memory B cell ELISpot. The autoantigen-specific spot-forming cells were presented as a concentration of positive spot-forming cells per 10^5^ B cells on the y-axis. The x-axis shows different groups. (C) Statistical analysis of total IgG secreting B cells in direct and memory B cell ELISpot. The total IgG spot-forming cells were presented as a concentration of positive spot-forming cells per 10^6^ PBMCs on the y-axis. The x-axis shows different groups. Data are presented as mean average ± SD for each of the antigens analyzed. ELISpot = B cell enzyme-linked immunospot, PBMC = peripheral blood mononuclear cell, SD = standard deviation.

B cell ELISpot was used to determine whether these autoantibodies were produced by specific autoreactive B cells *in vivo*. For all three groups (healthy control, HIV patients without treatment, and HIV patients on ART), B cell ELISpot assay was performed for 5 PBMCs samples (Figure 3A–C). In mixed autoantigen coated PVDF membrane wells, visualization of spots suggested the existence of autoantigen-specific B cells as Figure 3A indicated. The strongest autoantigen specific B cell response was observed in ART-naive HIV patient’ samples, and healthy control samples had the weakest response (Figure 3A, B). These results were consistent with the frequency of elevated autoantibodies in these 3 groups; the maximum prevalence of elevated autoantibodies was in ART-naive HIV patients, followed by HIV patients on ART (Table 1). Notably, healthy controls not only had a very low specific B cell response against autoantigens (Figure 3A, B), but also had relatively low total IgG production by B cells compared to HIV patients (Figure 3C), suggesting abnormal B cell activity in HIV patients.

MDSCs (originated from CD33^+^CD11b^+^) and Tregs (CD3^+^CD4^+^CD25^+^FoxP3^+^) play critical roles in the regulation of autoimmune responses. To examine which cell types are involved in the alterations of the autoantibody profile in HIV patients, we depleted the CD33^+^CD11b^+^ or CD3^+^CD4^+^CD25^+^ cells by FACS and performed B cell ELISpot assays on these depleted PBMCs. The depletion of CD3^+^CD4^+^CD25^+^ cells had no obvious effects on the production of autoantibodies or total IgG *in vitro* (Figure 3A, B), suggesting that Tregs do not play a significant role in autoantibody production by B cells *in vitro*. However, the depletion of CD33^+^CD11b^+^ cells remarkably impaired the production of both autoantibodies and total IgG in all 3 groups as shown in Figure 3. In order to further determine which subpopulation of CD33^+^CD11b^+^ cells contributes to this effect, CD33^+^CD11b^+^ cells were subsequently sorted into CD33^+^CD11b^+^HLA-DR^+^ cells and CD33^+^CD11b^+^HLA-DR^−^ cells (mainly M-MDSCs). These cells were cocultured with CD33^+^CD11b^+^ cell-depleted PBMCs at indicated ratios (Figure 3A, B). Interestingly, we observed that the CD33^+^CD11b^+^HLA-DR^+^ cells significantly promoted the production of both autoantibodies and total IgG in a dose-dependent manner, while the CD33^+^CD11b^+^HLA-DR^−^ cells had no effect (Figure 3A–C). These data suggest that the CD33^+^CD11b^+^HLA-DR^+^ cells rather than the CD33^+^CD11b^+^HLA-DR^−^ cells promote antibody production. Notably, the CD33^+^CD11b^+^HLA-DR^+^ cells showed similar effects on increasing antibody production in both healthy controls and HIV patients, which suggests that these cells play a role in boosting B cell response.

### Dramatic Elevation of CD33^+^CD11b^+^HLA-DR^+^ Cells in the Peripheral Blood of ART-Naive HIV Patients

**FIGURE 4:**
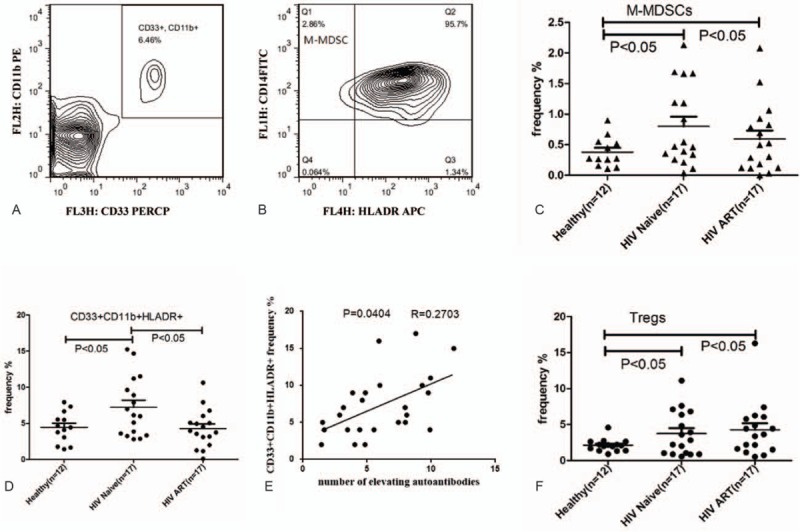
Frequency of CD33^+^CD11b^+^ cells, monocytic MDSCs, granulocytic MDSCs, CD33^+^CD11b^+^HLA-DR^+^ cells, and Tregs in peripheral blood of healthy controls and HIV infected individuals. (A, B) Gating strategy of M-MDSCs and G-MDSCs by flow cytometric analysis. CD11b^+^CD33^+^/high cells were first selected from live PBMCs (A), and the CD14^+^/HLA-DR^−^/low population as M-MDSC (B). (C, D, F) Comparison of M-MDSCs, CD33^+^CD11b^+^HLA-DR^+^ cells, and Tregs between HIV-infected ART-naive individuals, healthy controls, and HIV-infected individuals on ART. ^∗^*P* < 0.05: statistically significant difference from controls. (E) Correlation between CD33^+^CD11b^+^ cells frequencies and signal intensity of 12 autoantibody titers by microarray (NFI) (*r*^2^ = 0.2703, *P* = 0.0404; linear regression). ART = antiretroviral therapy, HIV = human immunodeficiency virus, MDSC = myeloid-derived suppressor cell, NFI = net fluorescent intensity.

To evaluate the correlation between the quantity of CD33^+^CD11b^+^HLA-DR^+^ cells and the alteration of the autoantibody spectrum, we compared the frequency of M-MDSCs (CD33^+^CD11b^+^HLA-DR^−^) cells, CD33^+^CD11b^+^HLA-DR^+^ cells, and Tregs (CD3^+^CD4^+^CD25^+^FoxP3^+^) in PBMCs from HIV-seropositive subjects (n = 33) with healthy subjects (n = 12). The CD33^+^CD11b^+^HLA-DR^+^ cells, accounting for nearly 90% of the CD33^+^CD11b^+^ cell population, in ART-naive HIV patients had a significantly higher frequency than both healthy controls and HIV patients on ART (*P* < 0.01), as shown in Figure 3. For the CD33^+^CD11b^+^HLA-DR^−^ cells, over 95% are CD14^+^ cells, namely M-MDSCs (M-MDSCs gate, Figure 4). When compared to healthy controls, the frequency of both M-MDSCs and Tregs were significantly elevated in ART-naive HIV patients or HIV patients on ART, which is consistent with previous reports.^12,18^ However, no significant difference in the frequency of M-MDSCs and Tregs was noted between these two HIV patient groups (ART-naive or on ART, *P* > 0.05).

Correlational analysis was performed between cell frequency and the occurrence of elevated autoantibodies in the available samples (from both healthy controls and HIV patients). Notably, only the frequency of the CD33^+^CD11b^+^ cells, or more specifically the CD33^+^CD11b^+^HLA-DR^+^ cells, was significantly correlated with the occurrence of elevated autoantibodies (*P* < 0.05) (Figure 4). These results support the conclusion that the CD33^+^CD11b^+^HLA-DR^+^ cells play an important role in the autoimmune manifestation caused by HIV infection, while the M-MDSCs and Tregs have no direct association with the emergence of autoantibodies in HIV patients. In order to understand the roles of CD33^+^CD11b^+^HLA-DR^+^ cells in the disease progression, we further performed correlational analysis between CD4 count and CD33^+^CD11b^+^ HLA-DR^+^ cells or elevated autoantibodies. In the HIV patients on ART, there was little correlation between CD4 count and the frequency of autoantibodies or the frequency of CD33^+^CD11b^+^HLA-DR^+^ cells. In the ART-naive HIV patients, a weak correlation (*P* = 0.0486, *R* = 0.235) between CD4 count and the frequency of CD33^+^CD11b^+^HLA-DR^+^ cells was identified (Supplement Figure 1). Additionally, although no significant correlation between CD4 and autoantibodies was observed in ART-naive HIV patients, most patients with extremely low CD4 counts (<300 cells/μL) displayed more than 8 elevated autoantibodies (Supplement Table 1).

## DISCUSSION

Autoimmunity has long been recognized to be associated with HIV infections. However, the complex nature of the mechanism of the autoimmune response *in vivo*, and minimal number of studies characterizing the spectrum of plasma autoantibodies in HIV patients in the presence or absence of ART implied that emerging autoantibodies in primary HIV patients is nonspecific.^1–3^ Therefore, it is crucial to evaluate whether there is a specific autoantibody profile related to HIV infection. In this study, we identified emerging autoantibody profiles in respect to HIV infections from 3 observations. First, altered autoantibody profiles were observed in ART-naive HIV patients and HIV patients on ART compared to healthy controls. Fifteen autoantibodies in ART-naive HIV patients and 8 of 15 in HIV patients on ART were significantly higher than that in healthy controls. Meanwhile, 5 of 15 autoantibodies in ART-naive HIV patients were significantly higher than that of HIV patients on ART. Additionally, the highest titer of the 15 specific elevated autoantibodies was also observed in ART-naive HIV patients. This study not only characterizes the spectrum of the plasma autoantibody profile, but also reveals the possibility of ART improving the autoimmune manifestation by decreasing the titer and incidence of autoantibodies in HIV patients.

Second, autoantibodies also displayed a significantly higher incidence in HIV patients than healthy controls. Particularly, the higher incidence of 15 specific autoantibodies is 2–10/17 in ART-naive HIV patients and 0–4/20 in healthy controls. Statistical analysis also confirmed that these 15 specific autoantibodies had a significantly higher prevalence in HIV patients (both ART-naive patients and patients on ART) than healthy controls. Finally, autoantibody-producing B cells were confirmed by B cell ELISpot. Similar to the order of interaction of autoantibodies from the 3 groups against autoantigens, the strongest autoreactive B cell response was found in the ART-naive HIV group, followed by HIV patients on ART, and healthy controls had the weakest response. Collectively, we have established the autoantibody spectrum related to HIV infection for the first time, and our data suggest that ART improves the autoimmune response during HIV infection.

Although some aspects of HIV immunopathogenesis relevant to the promotion of autoimmunity were discussed previously, the mechanisms underlying the interplay between immune dysregulation and the impact of ART may provide important insight into understanding the manifestations of autoimmunity during HIV infection.^26–32^ Immune dysregulation is a hallmark of HIV infection, which may be caused by altering the frequency of function of immune cells such as T cells, B cells, and other immune cells.^27–35^ Recent studies indicated that elevated immune regulatory cells (MDSCs and Tregs) are associated with progression of HIV/AIDS.^12–15^ Despite the lack of common biomarkers, MDSCs are believed to be derived from CD33^+^CD11b^+^ cells. Our *in vitro* studies demonstrated that depletion of CD33^+^CD11b^+^ cells, but not CD3^+^CD4^+^CD25^+^ cells leads to a remarkably impaired B cell response. As MDSCs encompass a heterogeneous population, CD33^+^CD11b^+^ cells strongly promote the B cell response *in vitro*. In these cells, more than 90% of the cells are CD14 positive and/or HLA-DR positive and <10% are MDSCs. Unexpectedly, additional experiments with sorted CD33^+^CD11b^+^ cells indicated that CD33^+^CD11b^+^HLA-DR^−^ cells (mainly MDSCs) have no effect on secretion of autoantibodies or total IgG *in vitro*. On the contrary, CD33^+^CD11b^+^HLA-DR^+^ cells promoted a robust production of both autoantibodies and total IgG in a dose-dependent manner. Notably, CD33^+^CD11b^+^HLA-DR^+^ cells displayed the same role in HIV patients and healthy controls, which suggest that CD33^+^CD11b^+^HLA-DR^+^ cells contribute to the development of autoimmunity in a nonspecific way.

Based on the previously mentioned results, we proposed that an increase in the CD33^+^CD11b^+^HLA-DR^+^ cells would promote the production of autoantibodies *in vivo*. Flow cytometry data from all participants displayed a significant increase in the frequency of CD33^+^CD11b^+^HLA-DR^+^ cells in ART-naive HIV patients rather than HIV patients on ART or healthy controls. Meanwhile, the frequency of CD33^+^CD11b^+^HLA-DR^+^ cells positively correlated with the occurrence of elevated autoantibodies per sample in both healthy controls and HIV patients. The above results suggest that a higher CD33^+^CD11b^+^HLA-DR^+^ cell frequency promotes increased production of elevated autoantibodies in ART-naive HIV patients, and ART can improve autoimmunity in HIV patients, which is at least partly due to decreased frequency of CD33^+^CD11b^+^HLA-DR^+^ cells.

Although MDSCs and Tregs have been documented to a play critical role in immune tolerance and the control of the autoimmune response, the effects of MDSCs and Tregs may be dependent on the interaction between these immune regulatory cells and T cells.^10–12,32–35^ Notably, T cell exhaustion and impaired function caused by HIV infection may hinder the function of these immune regulatory cells against the development of autoimmunity. On the contrary, immune activation and immune dysfunction lead to increased CD33^+^CD11b^+^HLA-DR^+^ cells, which promote the development of autoimmunity in a nonspecific way. The proposed mechanism of CD33^+^CD11b^+^HLA-DR^+^ cells in the promotion of the autoimmune response may shed light on these controversies in HIV infection and pathogenesis; an increased population of CD33^+^CD11b^+^ cells promotes persistent immune activation and autoimmune response, while an increased population of suppressor cells may lead to impaired T cell immunity.

In this study, we reveal that HIV infection leads to autoimmune manifestation with a specific spectrum of circulating autoantibodies, meanwhile ART may improve the autoimmune condition and alter their autoantibody spectrum by reducing the number of CD33^+^CD11b^+^HLA-DR^+^ cells. However, a better understanding of the mechanism behind the effect of ART on the CD33^+^CD11b^+^HLA-DR^+^ cells may provide the necessary insight for resolving the mechanism of immune dysfunction caused by HIV infection and reconstitution of the immune system in HIV patients, which requires further investigation.

## Supplementary Material

Supplemental Digital Content

## References

[1] Zandman-Goddard G Shoenfeld Y HIV and autoimmunity. *Autoimmun Rev* 2002; 1:329–337. 12848988 10.1016/s1568-9972(02)00086-1

[2] Kulthanan K Jiamton S Omcharoen V Autoimmune and rheumatic manifestations and antinuclear antibody study in HIV-infected Thai patients. *Int J Dermatol* 2002; 41:417–422. 12121558 10.1046/j.1365-4362.2002.01529.x

[3] Massabki PS Accetturi C Nishie IA Clinical implications of autoantibodies in HIV infection. *AIDS* 1997; 11:1845–1850. 9412703 10.1097/00002030-199715000-00009

[4] Savige JA Chang L Horn S Anti-nuclear, anti-neutrophil cytoplasmic and anti-glomerular basement membrane antibodies in HIV-infected individuals. *Autoimmunity* 1994; 18:205–211. 7858105 10.3109/08916939409007997

[5] Gonzalez CM Lopez-Longo FJ Samson J Antiribonucleoprotein antibodies in children with HIV infection: a comparative study with childhood-onset systemic lupus erythematosus. *AIDS Patient Care STDS* 1998; 12:21–28. 11361881 10.1089/apc.1998.12.21

[6] Williams WM Whalley AS Comacchio RM Correlation between expression of antibodies to histone H2B and clinical activity in HIV-infected individuals. *Clin Exp Immunol* 1996; 104:18–24. 8603524 10.1046/j.1365-2249.1996.d01-633.x PMC2200381

[7] Grunewald T Burmester GR Schuler-Maue W Anti-phospholipid antibodies and CD5^+^ B cells in HIV infection. *Clin Exp Immunol* 1999; 115:464–471. 10193419 10.1046/j.1365-2249.1999.00828.x PMC1905258

[8] Coll J Palazon J Yazbeck H Antibodies to human immunodeficiency virus (HIV-1) in autoimmune diseases: primary Sjogren's syndrome, systemic lupus erythematosus, rheumatoid arthritis and autoimmune thyroid diseases. *Clin Rheumatol* 1995; 14:451–457. 7586984 10.1007/BF02207681

[9] Stimmler MM Quismorio FP Jr McGehee WG Anticardiolipin antibodies in acquired immunodeficiency syndrome. *Arch Intern Med* 1989; 149:1833–1835. 2504122

[10] Chevalier MF Weiss L The split personality of regulatory T cells in HIV infection. *Blood* 2013; 121:29–37. 23043072 10.1182/blood-2012-07-409755

[11] Luan Y Mosheir E Menon MC Monocytic myeloid-derived suppressor cells accumulate in renal transplant patients and mediate CD4 (^+^) Foxp3 (^+^) Treg expansion. *Am J Transplant* 2013; 13:3123–3131. 24103111 10.1111/ajt.12461

[12] Qin A Cai W Pan T Expansion of monocytic myeloid-derived suppressor cells dampens T cell function in HIV-1-seropositive individuals. *J Virol* 2013; 87:1477–1490. 23152536 10.1128/JVI.01759-12 PMC3554138

[13] Garg A Spector SA HIV type 1 gp120-induced expansion of myeloid derived suppressor cells is dependent on interleukin 6 and suppresses immunity. *J Infect Dis* 2014; 209:441–451. 23999600 10.1093/infdis/jit469 PMC3883171

[14] Gama L Shirk EN Russell JN Expansion of a subset of CD14highCD16neg CCR2low/neg monocytes functionally similar to myeloid-derived suppressor cells during SIV and HIV infection. *J Leukoc Biol* 2012; 91:803–816. 22368280 10.1189/jlb.1111579 PMC3336772

[15] Weiss L Donkova-Petrini V Caccavelli L Human immunodeficiency virus-driven expansion of CD4^+^CD25^+^ regulatory T cells, which suppress HIV-specific CD4 T-cell responses in HIV-infected patients. *Blood* 2004; 104:3249–3256. 15271794 10.1182/blood-2004-01-0365

[16] Moreno-Fernandez ME Presicce P Chougnet CA Homeostasis and function of regulatory T cells in HIV/SIV infection. *J Virol* 2012; 86:10262–10269. 22811537 10.1128/JVI.00993-12 PMC3457299

[17] Torgerson TR Ochs HD Regulatory T cells in primary immunodeficiency diseases. *Curr Opin Allergy Immunol* 2007; 7:515–521. 10.1097/ACI.0b013e3282f1a27a 17989528

[18] Vollbrecht T Stirner R Tufman A Chronic progressive HIV-1 infection is associated with elevated levels of myeloid-derived suppressor cells. *AIDS* 2012; 26:F31–F37. 22526518 10.1097/QAD.0b013e328354b43f

[19] Greten TF Manns MP Korangy F Myeloid derived suppressor cells in human diseases. *Int Immunopharmacol* 2011; 11:802–807. 21237299 10.1016/j.intimp.2011.01.003 PMC3478130

[20] Gabrilovich DI Nagaraj S Myeloid-derived suppressor cells as regulators of the immune system. *Nat Rev Immunol* 2009; 9:162–174. 19197294 10.1038/nri2506 PMC2828349

[21] Nagaraj S Gabrilovich DI Myeloid-derived suppressor cells in human cancer. *Cancer J* 2010; 16:348–353. 20693846 10.1097/PPO.0b013e3181eb3358

[22] Hoechst B Ormandy LA Ballmaier M A new population of myeloid-derived suppressor cells in hepatocellular carcinoma patients induces CD4 (^+^)CD25 (^+^) Foxp3 (^+^) T cells. *Gastroenterology* 2008; 135:234–243. 18485901 10.1053/j.gastro.2008.03.020

[23] Liu CY Wang YM Wang CL Population alterations of L-arginase- and inducible nitric oxide synthase-expressed CD11b^+^/CD14/CD15^+^/CD33^+^ myeloidderived suppressor cells and CD8^+^ T lymphocytes in patients with advanced-stage nonsmall cell lung cancer. *J Cancer Res Clin Oncol* 2009; 136:35–45. 19572148 10.1007/s00432-009-0634-0 PMC11827779

[24] Li QZ Zhou J Wandstrat AE Protein array autoantibody profiles for insights into systemic lupus erythematosus and incomplete lupus syndromes. *Clin Exp Immunol* 2007; 147:60–70. 17177964 10.1111/j.1365-2249.2006.03251.x PMC1810453

[25] Olsen NJ Li QZ Quan J Autoantibody profiling to follow evolution of lupus syndromes. *Arthritis Res Ther* 2012; 14:R174. 22838636 10.1186/ar3927 PMC3580568

[26] Dhasmana DJ Dheda K Ravn P Immune reconstitution inflammatory syndrome in HIV-infected patients receiving antiretroviral therapy: pathogenesis, clinical manifestations and management. *Drugs* 2008; 68:191–208. 18197725 10.2165/00003495-200868020-00004

[27] Cohen S Shachar I Cytokines as regulators of proliferation and survival of healthy and malignant peripheral B cells. *Cytokine* 2012; 60:13–22. 22784632 10.1016/j.cyto.2012.06.019

[28] Tanaka T Kishimoto T Targeting interleukin-6: all the way to treat autoimmune and inflammatory diseases. *Int J Biol Sci* 2012; 8:1227–1236. 23136551 10.7150/ijbs.4666 PMC3491446

[29] Rickert RC Jellusova J Miletic AV Signaling by the tumor necrosis factor receptor superfamily in B-cell biology and disease. *Immunol Rev* 2011; 244:115–133. 22017435 10.1111/j.1600-065X.2011.01067.x PMC3202302

[30] Ohl K Tenbrock K Inflammatory cytokines in systemic lupus erythematosus. *J Biomed Biotechnol* 2011; 2011:432595. 22028588 10.1155/2011/432595 PMC3196871

[31] Mountz JD Wang JH Xie S Cytokine regulation of B-cell migratory behavior favors formation of germinal centers in autoimmune disease. *Discov Med* 2011; 11:76–85. 21276413 PMC3249418

[32] Stratton R Slapak G Mahungu T Autoimmunity and HIV. *Curr Opin Infect Dis* 2009; 22:49–56. 19532080 10.1097/QCO.0b013e3283210006

[33] Lane HC Masur H Edgar LC Abnormalities of B-cell activation and immunoregulation in patients with the acquired immunodeficiency syndrome. *N Engl J Med* 1983; 309:453–458. 6224088 10.1056/NEJM198308253090803

[34] Swingler S Zhou J Swingler C Evidence for a pathogenic determinant in HIV-1 Nef involved in B-cell dysfunction in HIV/AIDS. *Cell Host Microbe* 2008; 4:63–76. 18621011 10.1016/j.chom.2008.05.015 PMC2911124

[35] Hazenberg MD Otto SA van Benthem BH Persistent immune activation in HIV-1 infection is associated with progression to AIDS. *AIDS* 2003; 17:1881–1888. 12960820 10.1097/00002030-200309050-00006

