# Supplementary material for: Antiretroviral Therapy Normalizes Autoantibody Profile of HIV Patients by Decreasing CD33+CD11b+HLA-DR+ Cells: A Cross-Sectional Study
Source: Medicine (Baltimore). 2016 Apr 18;95(15):e3285. doi: 10.1097/MD.0000000000003285 (PMC4839811; doi:10.1097/MD.0000000000003285)

Supplement Table1. Profile of HIV-1-infected patients and elevated autoantibodies.

| Patient ID | Age (years) | Sex (M/F) | Race | Ethnicity | CD4 T cell (count/μl) | Plasma HIV RNA (copies/ml) | Elevated autoantibodies |
| --- | --- | --- | --- | --- | --- | --- | --- |
| G001 | 27 | M | BLACK | NH | 582 | 22,000 | 7 |
| G002 | 33 | M | CAUCASIAN | NH | 709 | 58,000 | 9 |
| G003 | 29 | M | CAU / Asian | NH | 233 | 140,000 | 8 |
| G004 | 30 | F | BLACK | NH | 238 | 57,000 | 13 |
| G006 | 52 | F | BLACK | NH | 607 | 5,250 | 7 |
| G007 | 31 | M | BLACK | NH | 466 | 17,500 | 13 |
| G008 | 27 | F | BLACK | NH | 865 | 3,550 | 10 |
| G009 | 31 | M | BLACK | NH | 241 | 25,000 | 10 |
| G010 | 27 | M | CAUCASIAN | NH | 809 | 99,900 | 10 |
| G011 | 28 | M | BLACK | NH | 321 | 85,800 | 6 |
| G012 | 20 | M | BLACK | NH | 239 | 45,000 | 8 |
| G013 | 25 | M | BLACK | NH | 445 | 348,266 | 10 |
| G014 | 22 | F | BLACK | NH | 591 | 240,000 | 5 |
| G015 | 18 | M | BLACK | NH | 247 | 23,000 | 9 |
| G016 | 20 | M | BLACK | NH | 362 | 36,000 | 3 |
| G017 | 27 | M | BLACK | NH | 233 | 140,000 | 8 |
| G018 | 33 | M | CAUCASIAN | NH | 709 | 58,000 | 5 |


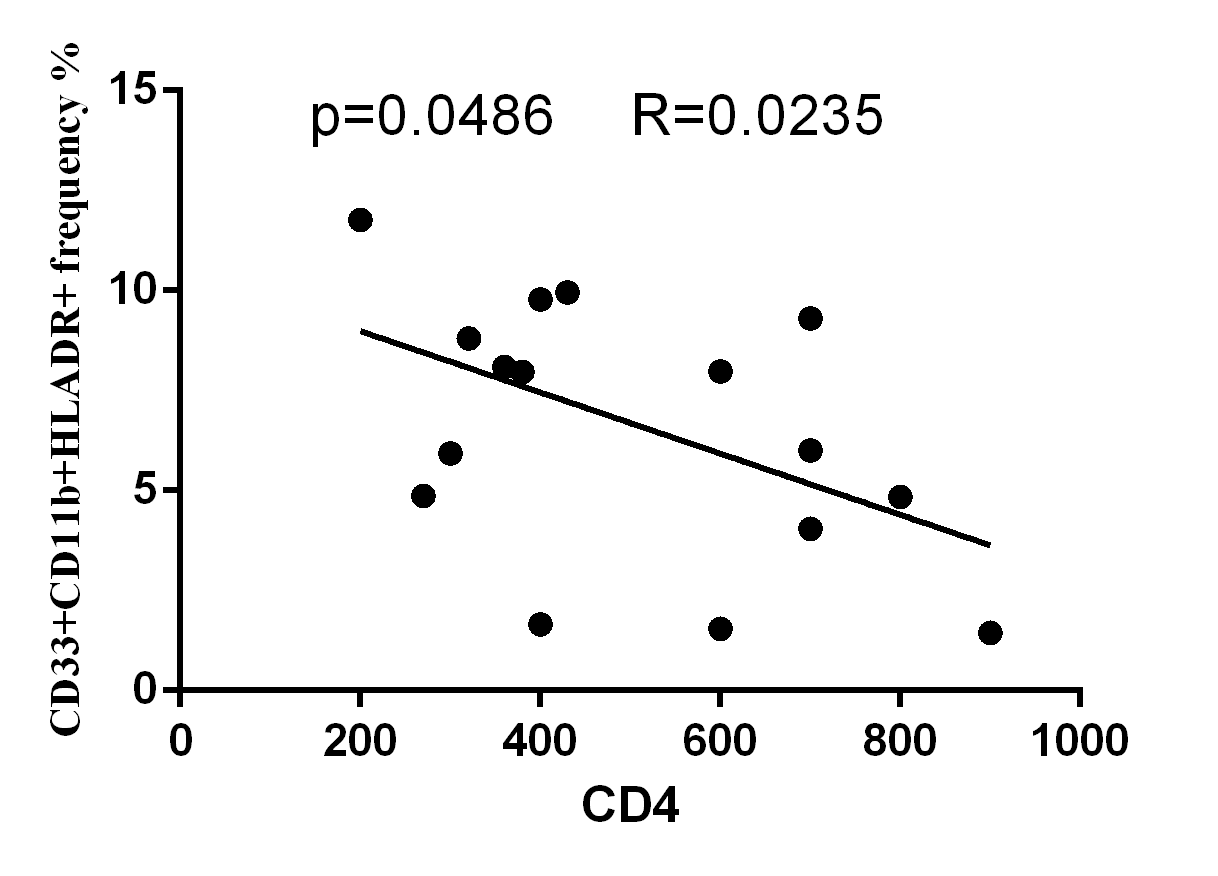

Supplement: Supplemental Digital Content [file medi-95-e3285-s001.doc]
